# *In Vivo* Chromatin Targets of the Transcription Factor *Yin Yang 2* in Trophoblast Stem Cells

**DOI:** 10.1371/journal.pone.0154268

**Published:** 2016-05-18

**Authors:** Raquel Pérez-Palacios, Sofía Macías-Redondo, María Climent, Bruno Contreras-Moreira, Pedro Muniesa, Jon Schoorlemmer

**Affiliations:** 1 Instituto Aragonés de Ciencias de la Salud and Instituto de Investigación Sanitaria de Aragón (IIS-Aragón), Zaragoza, Spain; 2 ARAID Foundation, Zaragoza, Spain; 3 Estación Experimental de Aula Dei /CSIC, Av. Montañana 1.005, 50059 Zaragoza, Spain; 4 Departamento de Anatomía, Embriología y Genética Animal, Facultad de Veterinaria, Universidad de Zaragoza, C/ Miguel Servet 177, 50013 Zaragoza, Spain; Wellcome Trust Centre for Stem Cell Research, UNITED KINGDOM

## Abstract

**Background:**

*Yin Yang 2* (YY2) is a zinc finger protein closely related to the well-characterized *Yin Yang 1* (YY1). YY1 is a DNA-binding transcription factor, with defined functions in multiple developmental processes, such as implantation, cell differentiation, X inactivation, imprinting and organogenesis. *Yy2* has been treated as a largely immaterial duplication of *Yy1*, as they share high homology in the Zinc Finger-region and similar if not identical *in vitro* binding sites. In contrast to these similarities, gene expression alterations in HeLa cells with attenuated levels of either *Yy1* or *Yy2* were to some extent gene-specific. Moreover, the chromatin binding sites for YY2, except for its association with transposable retroviral elements (RE) and Endogenous Retroviral Elements (ERVs), remain to be identified. As a first step towards defining potential *Yy2* functions matching or complementary to *Yy1*, we considered *in vivo* DNA binding sites of YY2 in trophoblast stem (TS) cells.

**Results:**

We report the presence of YY2 protein in mouse-derived embryonic stem (ES) and TS cell lines. Following up on our previous report on ERV binding by YY2 in TS cells, we investigated the tissue-specificity of REX1 and YY2 binding and confirm binding to RE/ERV targets in both ES cells and TS cells. Because of the higher levels of expression, we chose TS cells to understand the role of *Yy2* in gene and chromatin regulation. We used *in vivo* YY2 association as a measure to identify potential target genes. Sequencing of chromatin obtained in chromatin-immunoprecipitation (ChIP) assays carried out with αYY2 serum allowed us to identify a limited number of chromatin targets for YY2. Some putative binding sites were validated in regular ChIP assays and gene expression of genes nearby was altered in the absence of *Yy2*.

**Conclusions:**

YY2 binding to ERVs is not confined to TS cells. *In vivo* binding sites share the presence of a consensus binding motif. Selected sites were uniquely bound by YY2 as opposed to YY1, suggesting that YY2 exerts unique contributions to gene regulation. YY2 binding was not generally associated with gene promoters. However, several YY2 binding sites are linked to long noncoding RNA (*lncRNA*) genes and we show that the expression levels of a few of those are *Yy2*-dependent.

## Introduction

The *Yin Yang 2* (*Yy2*) gene is a retrotransposon-derived paralog of the *Yy1* (*Yin Yang 1*) gene, and is both conserved and active in all placental mammals [1]. In the mouse, the *Yy2* gene is localized on the X chromosome, where it is embedded in a complex *locus* shared with another gene, namely *Mbtps2* [2]. The *Yy2* gene encodes a 378 AA protein, which shares 56.2% identity overall with YY1. While the N-terminal region of YY2 is very different at the amino-acid level from the N-terminal region of YY1, the C-terminal region encoding four Gli-Kruppel type zinc finger domains is very well conserved (86.4% identity between YY1 and YY2). Consistent with the high level of sequence conservation, both YY1 and YY2 bind a consensus YY1 binding motif [3]. Similarly, largely identical motifs are bound by YY1 and YY2 when high affinity binding sites are selected for *in vitro* [1]. Moreover, competition between YY1 and YY2 for binding to virus-responsive binding sites has been proposed to underlie activation of the IFNβ gene [4]. Interestingly, *in vitro* binding assays also unveiled that YY1 and YY2 interact with RYBP and selected Polycomb group proteins [5].

YY1 is a transcription factor with sequence context-dependent activation or repression activity, which controls the transcription of a large number of viral and cellular genes [6]. Loss-of-function models have implicated YY1 in gene regulation underlying fundamental biological processes such as proliferation, cell cycle regulation and cytokinesis [7]. Considering all similarities between YY1 and YY2, functional redundancy has been implied. Nevertheless, the biological functions of YY2 have not been well characterized and loss-of-function models in the mouse are not available. Moreover, partial deficiency in HeLa cells revealed distinct *Yy1* or *Yy2*-dependent alterations in gene expression [8].

*In vivo*, YY1 and YY2 share DNA binding to transposable retroviral elements (RE) [9], particularly to selected members of the family of Endogenous retroviral elements (ERVs) [10]. ERV represent a category of repeat sequences that result from retroviral insertions in the germline. ERVs occupy 8–10% of mammalian genomes, and can be grouped into families based on sequence homology. Within each family, a multitude of very similar but distinct elements can be distinguished that are scattered across the genome. Most copies have accumulated mutations rendering them incompetent for infection and/or mobilization. Independent of their mobilization activities, ERVs impact the transcriptional activities of neighboring genes through promoter and long-range effects. ERV are active especially in germ cells, placenta and preimplantation embryos (reviewed in [11, 12]). Subtle differences in binding preference were obvious when comparing *in vivo* binding of YY1 family members to ERV elements [10].

During preimplantation development the first differentiation steps take place in the embryo, separating the inner cell mass (ICM) from the trophectoderm (TE), which give rise to the embryo proper and extra-embryonic tissues, respectively. Gene expression underlying these steps is governed in part by mechanisms unique to these particular stages and cell types [13, 14]. These include the maintenance of allele-specific expression of particular genes based on parental-origin specific methylation patterns (imprinting), and consecutive re-activation and inactivation of one of the X chromosomes in females (XCI). Both REX1 and YY1 are expressed during late preimplantation mouse development [15, 16]. YY1 is essential in this period, since *Yy1*-loss-of-function does not allow embryonic development beyond implantation [15]. YY1 also binds imprinted genes in a methylation-dependent way [17, 18] and both YY1 and REX1 contribute to XCI [18–20]. Although both YY1 and REX1 contribute to mammalian-specific processes such as embryo implantation, imprinting and XCI, such functions have not yet been described for *Yy2*. We considered the presence of YY2 in embryonic stem (ES) and trophoblast stem (TS) cells, as models for ICM and trophoblast precursors, respectively. We detected increased levels of *Yy2* in TS cells and decided to assess a regulatory role of YY2 in TS cells. In line with the apparent abundant presence of YY2 in TS cells, we initiated a search for genomic targets in addition to previously identified transposons. Binding sites identified were validated in ChIP assays and shown to uniquely bind YY2 as opposed to YY1. Moreover, several long noncoding RNAs (lncRNAs) linked to YY2 binding sites are moderately affected by *Yy2*-deficiency, suggesting YY2-dependent regulation.

## Materials and Methods

### Immunological reagents and Western blot

The αYY2 and the αREX1 sera raised in rabbit have been described previously [10]. The aminoterminal region of YY2 (aminoacids 1–173) used for immunization does not show much homology to the corresponding region in YY1 [1], and does not cross-react with YY1 (S2 and S3 Figs). The rabbit αYY2 serum was further affinity-purified over YY2α-GST protein (ImmunoStep SA, Salamanca, Spain). Monoclonal αHA (clone HA-7) was obtained as an unpurified ascites fraction (Sigma H9658).

For Western blot analysis, cells were washed twice with ice-cold PBS on the plate, scraped, resuspended in lysis buffer (0.25 M Tris–HCl (pH 7.8), 0.1% NP-40) containing protease inhibitors (PMSF, Sodium Ortovanadate and Complete; ROCHE) and protein stabilizers (DTT) as described [21]. Samples with added Laemmli sample buffer were subjected to SDS–polyacrylamide gel electrophoresis, transferred to nitrocellulose membranes (Hybond-P, Amersham). Standard Western blot was performed using αYY2, αREX1, αYY1 or αTUBULIN antibodies. Horseradish peroxidase-coupled αIgG secondary antibodies (Dako, α-mouse (1:2500), α-rabbit (1:10000)) were detected using chemiluminescence (ECL Western Blotting Detection, Amersham Biosciences). Before blocking and reprobing with a different primary antibody, membranes were stripped by incubation in 1 M glycine (pH 2.5) for 20 min at RT, followed by incubation in Tris-HCl pH 7.4 and TBS (50 mM Tris-Cl, pH 7.5; 150 mM NaCl) for 1 minute each.

### Indirect immunofluorescence and confocal microscopy

Cells were cultured on gelatin-coated coverslips for at least 4 h to attach. Cells were fixed with 4% paraformaldehyde in PBS (pH 7.4), permeabilized in 0.5% Triton-X100 for 10 min and incubated overnight at 4°C with a rabbit αYY2 antibody (1:4800) in PGBA (0.1% gelatin, 1% BSA, 0.05% NaAzide in PBS, pH 7.4)/10% FBS. All further steps were performed at RT. Cells were washed three times and incubated with an α-rabbit biotin antibody (1:500) for 30 min and with streptavidin-Alexa 488 (1:200) for 45 min both in PGBA. Nuclei were counterstained with DAPI (10 μg/ml) in PBS for 10 min. Coverslides were mounted in glycerol/DABCO 2.5%/50 mM Tris pH 8.6 (DTG). Negative controls were performed using the same procedure without the addition of primary antibody. Widefield microscopy images were obtained in an Olympus upright fluorescence microscope (BX60) with 40X objective. Confocal sections were obtained in an Olympus confocal microscope Fluoview FV1000 with a 60X objective. Images were pseudocolored in green for YY2 and DAPI in blue (cell nuclei).

### Cell culture, differentiation, transfection and separation

The mouse TS cell line B7 [22] was maintained as described [23] on 1% gelatin-coated tissue culture dishes and the presence of 70TS-CM supplemented with FGF4 (25 ng/ml) (Peprotech) and heparin (1 mg/ml) (Sigma) [5]. ES cell line E14T [24] was maintained on gelatin-coated tissue culture dishes in medium supplemented with 10% fetal calf serum, leukemia inhibitory factor (LIF) as described [25]. E14T ES cells were transfected with plasmids expressing shRNAs, selected with Hygromycin for 7 days as described before [5], and processed for Western blot. Attenuation of *Yy2* levels in TSB7 cells was achieved by introduction of vectors that express short hairpin RNA (shRNA) and GFP [10]. shRNA sequences introduced in different vectors are as follows: sh*Yy2*: 5´ CAATACCACTCTCCTGTTATT; shControl: 5´ AAGCGCGATCACATGGTCCTG. A detailed description of sh*Yy2* sequences used for knockdown is available in a manuscript in preparation [26]. Cells seeded the day before transfection (0.8 ×10^6^ cells per 30 mm dish) were transfected with Lipofectamine 2000 according to manufacturer's instructions (Invitrogen), using 4 μg of the plasmid of interest as described previously [5]. Cells were dissociated 16–24 h after transfection, suspended in TS medium supplemented with 1% FBS and 10 mM HEPES (Sigma) (2 × 10^6^ cells/ml). Cell sorting was performed on a FACSAria (BD BioSciences) equipped with a 488 nm laser and operated by the Flow Cytometry and Cell Separation Unit at CIBA, IACS, Zaragoza. GFP+ and GPF- sorted cells were collected in PBS/10% FBS. Cells were spun down, lysed in TRIzol^®^ reagent (Invitrogen) and processed for qPCR analysis as described below.

### Gene expression analysis: RT-qPCR

Cells were washed with PBS, scraped and total RNA was extracted using TRIzol^®^ reagent (Invitrogen). After digestion of genomic DNA (RQ1 RNAse-Free DNase, Promega), RNA was extracted with phenol/chloroform, precipitated with ethanol, re-suspended in water and quantified using Nanodrop (Thermo Scientific). cDNA was synthesized from 2 μg of RNA with random hexamer primers (ThermoScript^®^ RT-PCR System, Invitrogen) and stored at -20°C until used. cDNA was analyzed by quantitative PCR (Platinum^®^ SYBR^®^ Green qPCR SuperMix-UDG, Invitrogen) on an ABI Prism 7000 Real-Time PCR system, reactions were performed in triplicate. To compare expression levels between cell types, data obtained in at least two (and in most cases three) independent samples were processed using the ΔΔCt method [27], using *Gapdh* as a reference gene. To compare the capacity of different primer pairs to amplify annotated transcripts in the *Mbtps2/Yy2* locus, ΔCt values using *Gapdh* as a reference gene were calculated. Sequences of all primers used are included in S1 Table. Standard curves of all primers (used at 200 nM) were performed to assure efficient amplification (between 90% and 110%). Melting curves were also performed to verify production of single DNA species with each primer pair, except for ERV sequences.

### Chromatin Immunoprecipitation (ChIP) and *locus*-specific PCR

ChIP assays were performed as described [5, 10] with the following minor modifications. 5 x 10^6^ cells for each experimental condition were chemically crosslinked for 15 min at room temperature by the addition of fresh 37% formaldehyde solution to a final concentration of 1% (v/v). After the addition of glycine to a final concentration of 125 mM to stop the crosslink, cells were rinsed twice with PBS and harvested by scraping. Cells were resuspended in lysis buffer (50mM Tris-HCl (pH 8.1), 10mM EDTA, 1% (w/v) SDS) and protease inhibitors (Complete^™^, Roche) and sonicated in a Diagenode sonicator (30 second pulses, 30 second pause between pulses). Per 5 × 10^6^ cells the following sera were used for immunoprecipitation (IP): preimmune serum (PreI) (75 μg), rabbit anti-REX1 IgG (75 μg), mouse monoclonal H-10 anti-YY1 (Santa Cruz, 2.5 μg), rabbit anti-YY2 (25 μg). We compared semi-quantitative PCR amplification on amounts of chromatin obtained from the same number of cells after immunoprecipitation using either PreI, αYY2 or αREX1 serum. PCR products were visualized using ethidium bromide and photographed. Primers used are listed in S1 Table.

Quantitative real-time PCR on ChIPs was performed in triplicate reactions as described above and processed using the ΔCt method. Amplification was performed in 15 μl reactions using the following parameters: 50°C for 2 min, 95°C for 10 min followed by 40 cycles of (95°C 15 s, 60°C 60 s). Data presented show the aggregate of a minimum of two (and often three) separate ChIPs performed on different days, unless indicated otherwise. Enrichment levels were calculated as a percentage of immunoprecipitation relative to the input as described (www.SABiosciences.com). Data are shown as fold enrichment of a particular *locus* compared to control *loci*.

### High-throughput sequencing

ChIPped DNA for high-throughput sequencing was obtained by standard ChIP experiments using αYY2 or PreI serum as described above. High-throughput sequencing was performed by Fasteris SA (CH-1228 Plan-les-Ouates, Switzerland) on an Illumina Hi-Seq 2000 instrument according to manufacturer provided protocols with minor modifications. The Burrows-Wheeler Alignment Tool (0.5.9) was used to map the reads (50 nt) against the mouse genome (Mus Musculus—Version mm9, NCBI assembly M37), only quality-filtered reads were retained for downstream analyses. Mapping was carried out allowing a maximum of 1 mismatch per read. Reads mapping to several positions on the reference genome with the same mapping quality were considered repeated (R), and assigned a location by randomization. Additional data related to each of the resulting datasets is shown in Table 1, specific ChIP using αYY2 was compared to ChIP using PreI serum.

**Table 1:** Sequencing data.

	αYY2	PreI
initial number of quality-filtered reads	14746400	23649193
number of mm9 mapped reads	13369042	21612138
% of mapped reads respect to the initial number	90.66%	91.39%
number of reads mapped at unique positions	11056749	18052709
% number of reads mapping at unique positions **	74.98%	76.34%
number of reads mapping at multiple positions	2312293	3559429
% of reads mapping at multiple positions	15.68%	15.05%

Sequencing results. High-throughput sequencing was carried out on DNA obtained from chromatin immunoprecipitations (ChIPs) carried out using either αYY2 or PreI serum. The table shows sequencing and mapping data (**, % relative to the number of mm9 mapped reads).

Mapping results were loaded into the software SeqMonk (Version V0.16.0, License GPLv3) to identify peaks. SeqMonk counts reads in fixed size windows of 100 bp (step size = 50 bp). Counts in the YY2 dataset were multiplied by 1.62 to normalize for the number of mm9-mapped reads in each dataset, and counts of 0 were substituted for 1 to allow the calculation of ratios. We assigned an arbitrary score to each peak identified, which was calculated as the number of reads in αYY2 IP divided by the number of reads in the PreI sample in the same area (unique and repeated reads combined). The peaks were then ranked from high to low numbers, and annotated with the closest gene or database feature (within a range of 100 kb). ChIPseq data are freely available at http://www.ncbi.nlm.nih.gov/geo/query/acc.cgi?acc=GSE80824. To find overrepresented sequence motifs, search programs [28] were accessed through http://rsat.ulb.ac.be/rsat. The sequences of the top 20 peaks were used as input for CONSENSUS [29]. The best obtained position weight matrix was found to be comparable to motifs produced by alternative software such as MEME [30].

## Results

### Expression of YY2 in blastocyst-derived stem cells

We obtained a rabbit αYY2 serum after immunization with an YY2-GST fusion protein (see M&M), which easily detects the fusion protein in Western blot (S1A Fig). Furthermore, the serum detects HA-YY2 in the nucleus of transfected 293T cells using immunofluorescence (S1B Fig). The serum allowed the detection of endogenous YY2 protein in extracts from mouse ES cells by Western blot (S1C Fig). The presence of the approximately 60 kD band corresponding to YY2 was substantially reduced in extracts from cells transfected with shRNAs directed against *Yy2* (S1C Fig). Epitope detection by the serum in Western blot was abolished after saturation with excess binding protein (S1A Fig), confirming the specificity of detection. We expected the serum to be specific for YY2 as the YY2 portion used for immunization shares little homology with YY1 (S2 Fig). This was confirmed by Western blot, as the YY2 serum used does not immunoprecipitate or detect HA-tagged YY1 in Western blot as opposed to HA-tagged YY2 (S3A and S3B Fig).

**Fig 1:**
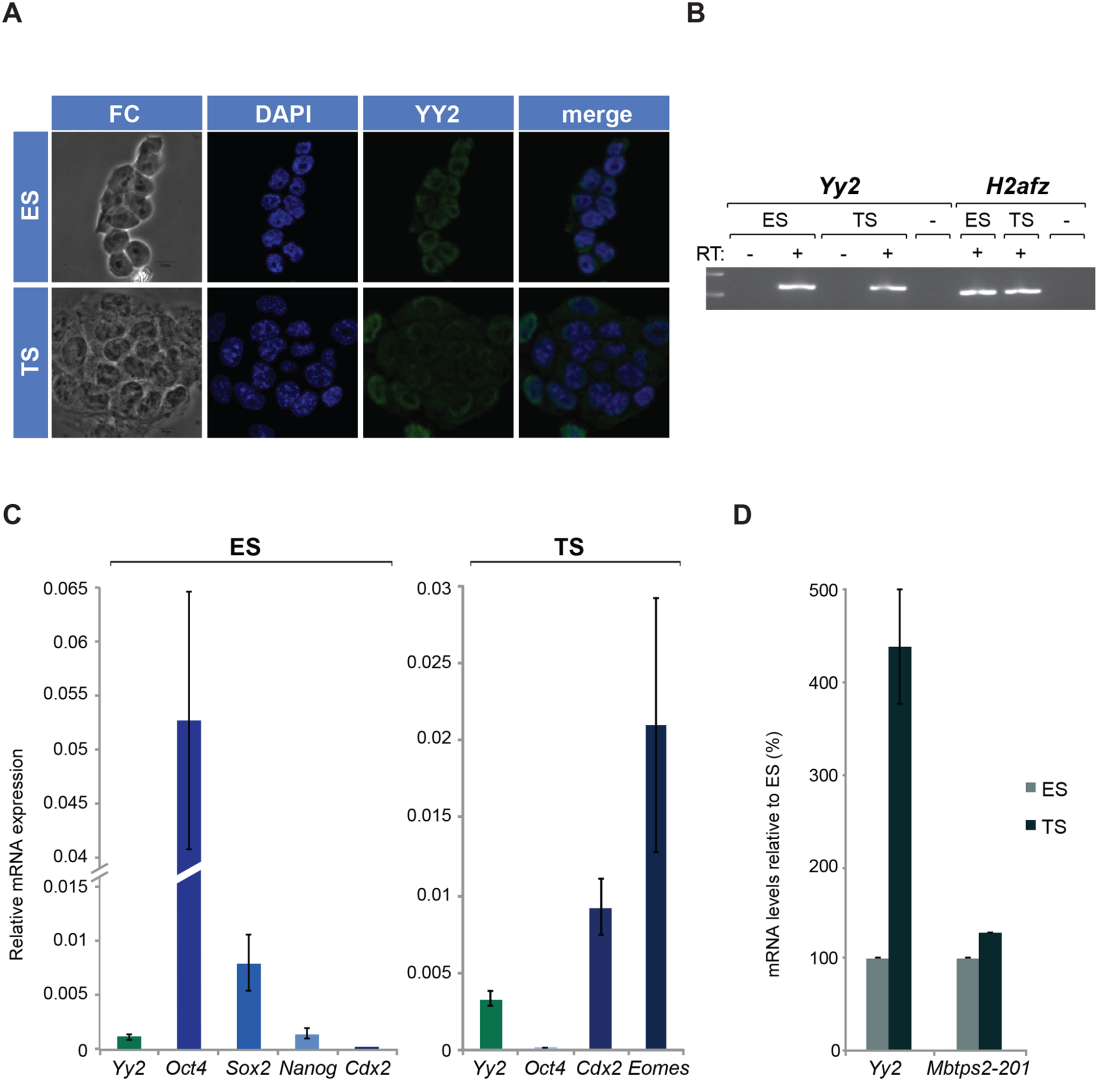
*Yy2* and *Mbtps2* expression in ES and TS cells. (A) Expression of YY2 was detected by indirect immunofluorescence in ES cells and TS cells. YY2 was visualized using αRabbitBiotin followed by StrepAlexa488, nuclei were stained with DAPI (blue). (B) An EtBr stained gel shows PCR products generated with either *Yy2* or *H2afz*-specific primers (as indicated), after amplification of cDNA obtained from ES and TS cells. (C) Expression of *Yy2* and cell type-specific markers (or lineage markers) of ES cells (*Oct4*, *Sox2*, *Nanog*) or TS cells (*Cdx2*, *Eomes*) was measured by RT-qPCR. Gene expression was normalized to *Gapdh* and data is represented as the mean ± SEM. (D) Expression of *Yy2* and *Mbtps2-001* was detected by quantitative RT-PCR in (E14T) ES cells or (TSB7) TS cells (error bars represent SEM). Transcript levels were normalized to *H2afz* and to the expression in ES cells (100%).

Although *Yy2* expression has been described as rather ubiquitous (although divergent levels have been reported [31]), its presence in preimplantation embryos or stem cells derived thereof has not been described so far. As *in vitro* models of either the ICM or the trophectoderm, we analyzed the presence of immuno-reactivity against the αYY2 serum in ES cells (line E14T) and TS cells (line B7) by indirect immunofluorescence. Staining patterns were analyzed by confocal microscopy and representative images are depicted in Fig 1A and S4 Fig. No staining was observed in either cell type when PreI serum or no primary antibody was used (data not shown). In ES cells, YY2 staining was apparent (panels YY2), mostly confined to the nucleus as revealed by nuclear counterstaining (panel DAPI). This pattern was confirmed by staining of HA-YY2 in the nucleus of transfected 293T cells (S1B Fig). Most if not all TS cells also showed detectable reactivity with the anti-YY2 serum (Fig 1A, panel TS). Similar to ES cells, staining was most intense in the nucleus. YY2 (green) displays a predominantly nuclear localization in ES and TS cells, although some weak staining was observed in the cytoplasm of TS cells.

To further confirm the presence of YY2 in ES and TS cells we also tested *Yy2* mRNA levels in blastocyst-derived stem cell lines in tissue culture by PCR. Expression of *H2Afz* was used as a control for the quality of the RNA and cDNA used. Both *H2afz* and *Yy2* mRNA were easily detected in both cell types (Fig 1B). *Yy2* transcript levels were analyzed by quantitative PCR and compared to the levels of several lineage-specific transcription factors. Expression of *Oct4*, *Sox2* and *Nanog* was confined to ES cells as expected (Fig 1C), and the converse was true for *Cdx2* and *Eomes*. *Yy2* was detected in ES cells at levels similar to *Nanog*. Relative to expression levels in ES cells (assigned arbitrarily a 100% level), we detected levels in TS cells at about 400% (Fig 1D).

Transcripts encoding YY2 are generated from a complex *locus* that also produces a variety of transcripts called *Mbtps2* [2]. The major transcript *Mbtps2-001* is expressed at comparable levels in both TS and ES cells (Fig 1D). The *Mbtps2 locus* produces various transcripts (S5A Fig), including several that overlap directly with *Yy2*. To discriminate properly between transcripts transcribed from this *locus* that encode either MBTPS2 or YY2, we extended our analysis to include the mRNA levels of several annotated *Mbtps2* variant transcripts using qPCR. Results (S5 Fig) demonstrate the presence in TS cells of both the canonical *Mbtps2-001* transcript and of a *Mbtps2-003* transcript. Although this transcript is named *Mbtps2-003*, it is identical to the *Yy2-201* transcript and the putative protein encoded in the 5´end is not represented in the protein database (Swiss Prot). In accordance with the hypothesis that the *Mbtps2-003* transcript is identical to the *Yy2-201* transcript, amplification efficiencies (Cts, S5B Fig) obtained using *Mbtps2-003*–specific primers (primer set D) are almost identical to those obtained using primers in the *Yy2* coding region (primer sets A, B; S5 Fig). Assays aimed at the detection of different exons of the *Mbtps2-002* transcripts that might overlap with the *Yy2 locus* (S5A Fig) yielded Ct values in a high range compatible with lack of expression (S5B Fig). Altogether, we conclude that the *Yy2* transcript levels measured (Fig 1D) can be attributed to YY2-encoding transcripts, without interference from transcription that covers the *Mbtps2* gene.

### REX1 and YY2 binding to ERVs of different families in different stem cells

**Fig 2:**
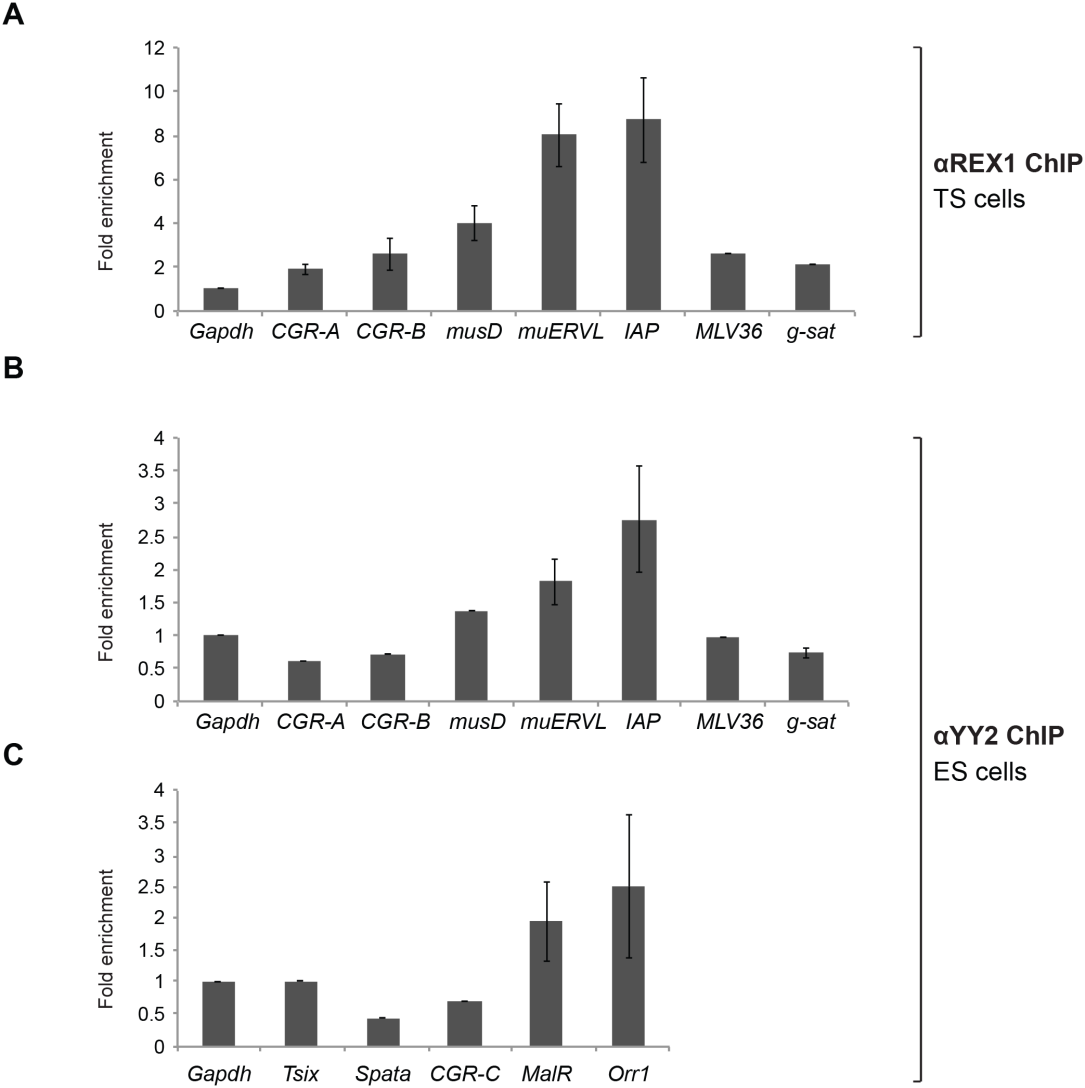
REX1 and YY2 bind RE/ERV elements in both ES and TS cells. (A) REX1 association to RE in TS cells. Binding was assessed in chromatin immunoprecipitation assays using αREX1 serum followed by quantification of precipitated DNA using real-time qPCR amplification. The figure shows analysis of non-binding reference sites as controls (CGR-A, CGR-B), and *Gapdh*, several sequences present in the genome as multiple copies (MLV36, γ-satellite) and the ERV elements indicated. Association is represented as percentage bound (relative to purified chromatin extract from the same lysate). Enrichment was calculated as percentage association relative to control chromatin, and is represented as fold binding or enrichment relative to a non-binding reference gene *Gapdh*. Error bars indicate SEM. (B) As A except for YY2 binding in ES cells. Data on CGR-A, CGR-B and *musD* are from a single experiment. (C) YY2 binding to Class III elements. Binding of YY2 in ES cells to class III ERV sequences present in the genome as multiple copies (*Orr1* and *MalR*) was assessed by qPCR analysis as described in **A**. The figure shows fold enrichment relative to a non-binding reference gene *Gapdh*. Data on CGR-C, *musD*, *Tsix* and *Spata* are from a single experiment.

Previously, we reported the association of REX1 (in ES cells) and YY2 (in TS cells) to several transposable retroviral elements [10], albeit with differing specificity of either protein for muERV-L and IAP elements, respectively. However, REX1 binding in ChIP assays may be tissue-specific [5]. To assure that the differences observed reflect binding specificities as opposed to cell type differences, we re-assessed binding of both factors to a subset of genomic repeats derived from LTR retrotransposons in ES and TS cells. As before, we carried out ChIP assays to demonstrate *in vivo* association of either REX1 or YY2 to a subset of ERV. After subtraction of background values and normalization against a control promoter, hardly any enrichment was observed for several control markers, i.e. different nonspecific genomic regions (CGR-A, CGR-B), or multi-copy sequences (MLV36, y-satellites) in both cases (Fig 2A and 2B).

We now assayed for REX1 binding in undifferentiated TS cells. In chromatin immunoprecipitated from TS cells using αREX1 (Fig 2A), we demonstrate a several-fold enrichment of both IAP and muERVL elements, and a weaker enrichment of musD elements. REX1 association to the same ERV was lower in TS cells (Fig 2) as opposed to ES cells [10, 32], in line with reduced expression of *Rex1* in TS cells [5]. In contrast to ES cells, enrichment for IAP elements was as high as for muERV-L (Fig 2A). Although the ratio of binding in TS cells relative to ES cells was slightly different for individual elements, REX1 associated to the same elements in both cell types.

YY2 associated with specific ERV elements in TS cells [10]. As YY2 was also detectable in ES cells (Fig 1), we re-assessed chromatin binding in these cells to assay for potential tissue-specific binding. Enrichment of a subset of ERVs in ChIP assays was also detected in ES cells. We observed a reproducible 3-fold enrichment of the IAP *locus* in chromatin immunoprecipitated using αYY2 in ES cells (Fig 2B). To a lesser extent, reproducible enrichment was also detected for musD and muERVL elements (Fig 2B). The association of YY2 to IAP was slightly stronger as compared to muERVL in both TS cells [10] and ES cells (Fig 2), indicating that the binding specificities of YY2 did not show cell type specificity. We also calculated a weak but reproducible 2-fold enrichment of the MalR and Orr1 *loci* in chromatin immunoprecipitated using αYY2 in ES cells (Fig 2C). By contrast, no reproducible enrichment was detected for *Tsix* and *Spata24*, *loci* efficiently bound by REX1 in ES cells [33]. In conclusion, YY2 associates with a subset of genomic repeats derived from LTR retrotransposons, displaying the highest affinity for IAP elements in both TS and ES cells. This result confirms our previous observation [10] that the *in vivo* association of YY2 to LTR retrotransposons is similar to but not identical to REX1.

### Identification of *in vivo* YY2 chromatin binding sites in TS cells

Taking into account that YY2 was easily detected in TS cells (Fig 1) and associated with specific ERV elements in these cells (Fig 2; [10]), we continued our functional analysis of *Yy2* in these cells. To identify YY2 binding sites in TS cell chromatin, ChIP experiments were carried out as before, and chromatin fractions obtained were sequenced (ChIPseq experiment, see M&M). YY2 binding sites were identified as regions with higher numbers of reads (peaks). A score based on the number of reads (see M&M) was assigned to each peak, and peaks were subsequently ranked from high to low numbers (Table 2). The total number of peaks identified with an enrichment score higher than 2 was 483 (data not shown), while 58 peaks scored above 4 (maximum 15). As these scores were relatively low, genome-wide coverage was probably not attained. Nevertheless, this procedure yielded a list of potential *in vivo* YY2 binding targets. In the absence of a biological replicate dataset, we validated YY2 binding to several of the putative binding sites.

**Table 2:** Putative *in vivo* YY2 binding sites.

Peak N°	Code	Chr	Location		Features	Description
1		17	4626100	4626650	U6.463	U6 spliceosomal RNA
2		4	146794200	146794350	Gm16889	predicted gene, 16889 Gene
3		4	145702600	145702850	Gm13247	predicted gene 13247 Gene
4	*T5T1*	5	92432350	92432500	Cdkl2	cyclin-dependent kinase-like 2 (CDC2-related kinase) Gene
5		3	66084950	66085100	Veph1	ventricular zone expressed PH domain homolog 1 (zebrafish) Gene
6		18	3009150	3009250	null	
7		6	144393150	144393300	Sox5	SRY-box containing gene 5 Gene
8		8	19932850	19933000	Gm10348	predicted gene 10348 Gene
9		7	101995950	101996050	null	
10		4	146017850	146017950	Gm13051	predicted gene 13051 Gene
11		13	65753950	65754100	AC116589.1	No description
12		5	96865250	96865400	Fras1	Fraser syndrome 1 homolog (human) Gene
13		7	97373550	97373700	Ccdc83	coiled-coil domain containing 83 Gene
14		8	9597100	9597250	Fam155a	family with sequence similarity 155, member A Gene
15	*T11T1*	11	19506300	19506400	Gm12027	predicted gene 12027 Gene
16		12	92957600	92957700	Ston2	stonin 2 Gene
17	*T13T1*	13	41048000	41048100	Gcnt2	glucosaminyl (N-acetyl) transferase 2, I-branching enzyme Gene
18		15	63758200	63758300	Fam49b	family with sequence similarity 49, member B Gene
19	*T18T1*	18	34326250	34326350	U7.84	U7 small nuclear RNA
20		14	20428900	20429000	Gm5458	predicted gene 5458 Gene

High-throughput sequencing was carried out on DNA obtained from chromatin immunoprecipitations (ChIPs) carried out with αYY2 serum. Reads were mapped to the mouse genome (Version mm9, NCBI assembly M37). The table shows data related to the 20 most significant peaks based on enrichment in the αYY2 dataset compared to the control dataset. The table lists the chromosomal localization of each peak, and the nearest annotated feature in the database within a range of 100 kb. Codes used to identify *loci* in ChIP assays are indicated as well. Note that several features in the table have changed names in the mm10 version of the mouse genome (NCBI GrCm38): Gm16889 has been renamed as *C230088H06-Rik* and Gm10348 has been replaced by first *Gm17279-201* and later *Gm26804-201*.

**Fig 3:**
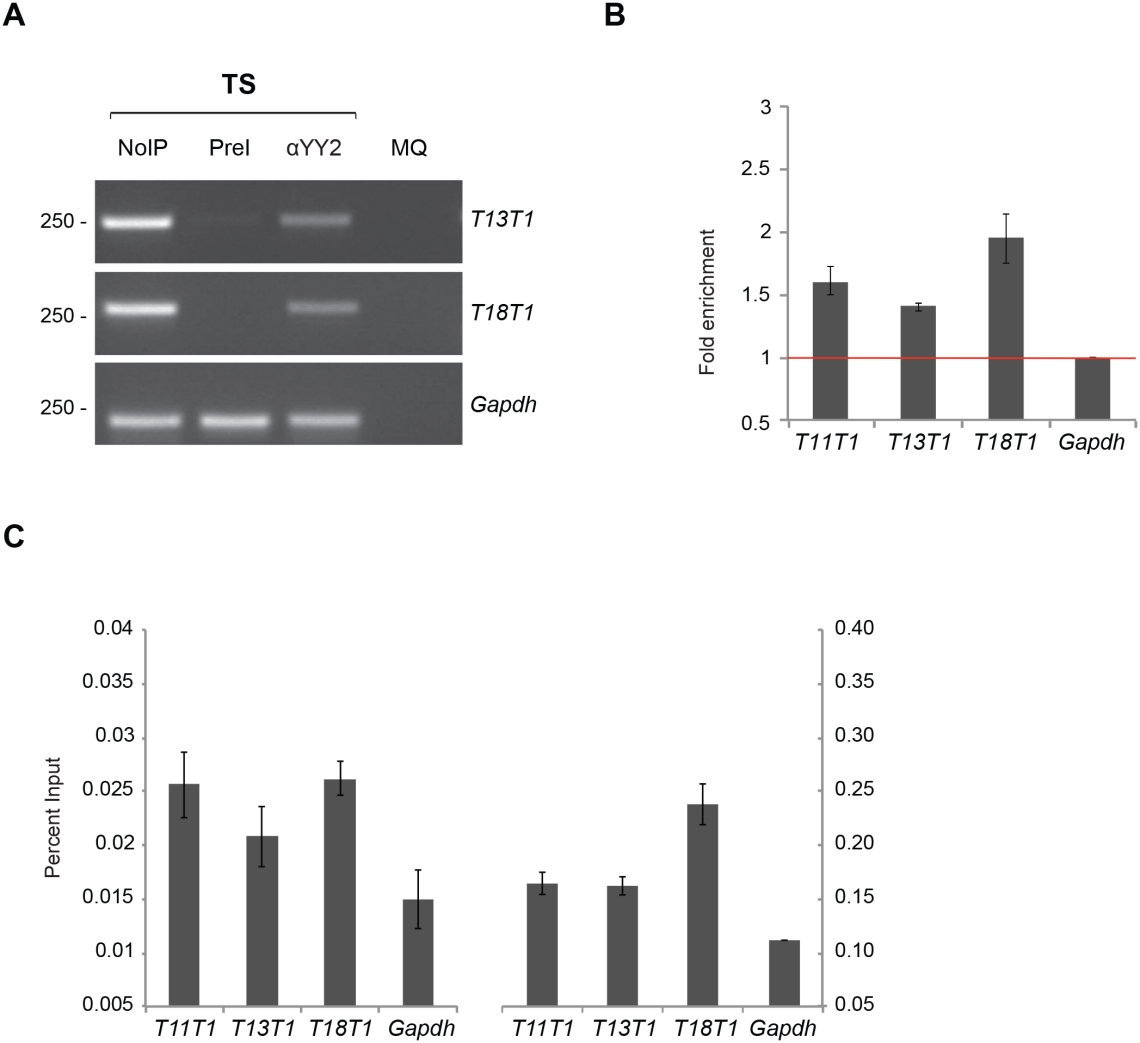
YY2 binding to genomic targets. (A) Semiquantitative PCR and (B) qPCR to validate YY2 association in TS cells to peaks identified by sequencing (Table 2). (A) EtBr stained gel shows PCR products of the genomic regions immunoprecipitated by YY2 (αYY2). Preimmune serum (PreI) was used as a control. Purified chromatin extract from the lysate was used to confirm amplicons. MQ, reactions without input DNA served as a negative control. *Gapdh* promoter was used as a reference gene. (B) Association of YY2 in TS cells to potential genomic targets (Table 2) was assessed by *locus*-specific qPCR analysis after chromatin immunoprecipitation using αYY2, or preimmune serum (PreI) as a control. The *Gapdh* promoter is included as a negative control. The amount of immunoprecipitated DNA as a percentage of input DNA was recalculated as fold association normalized to the *Gapdh* promoter. Error bars represent SEM. (C) As B, data is represented directly as a percentage of input DNA. Each panel represents an independent experiment.

As binding *loci* did not correspond to genes in a straightforward pattern, we assigned codes to binding sites (Table 2) without references to adjacent genes. When association was assayed using semi-quantitative PCR (Fig 3A), efficient amplification was observed using control templates for each of the *loci* analyzed (lanes NoIP), and hardly any amplification was observed for the majority of markers with PreI serum (lanes PreI). By contrast, we observed reproducible association of YY2 to the *T13T1* and *T18T1 loci*, as opposed to an intergenic fragment in chromosome 6 (data not shown) and the *Gapdh* promoter (Fig 3A), which were not amplified above background. Enrichment of binding sites in ChIP assays was also measured by qPCR. We calculated a reproducible 2 fold enrichment of the *T18T1 locus* in chromatin immunoprecipitated using αYY2 in TS cells (Fig 3B). Weak but reproducible enrichment was also detected for *T11T1* and *T13T1* sites (Fig 3B). This enrichment was observed irrespective of its representation as fold enrichment (Fig 3B) or as percentage of input chromatin (Fig 3C). These results validate the peaks identified as putative YY2 binding sites in TS cells (Table 2).

### Absence of YY1 association to *in vivo* YY2 binding sites

**Fig 4:**
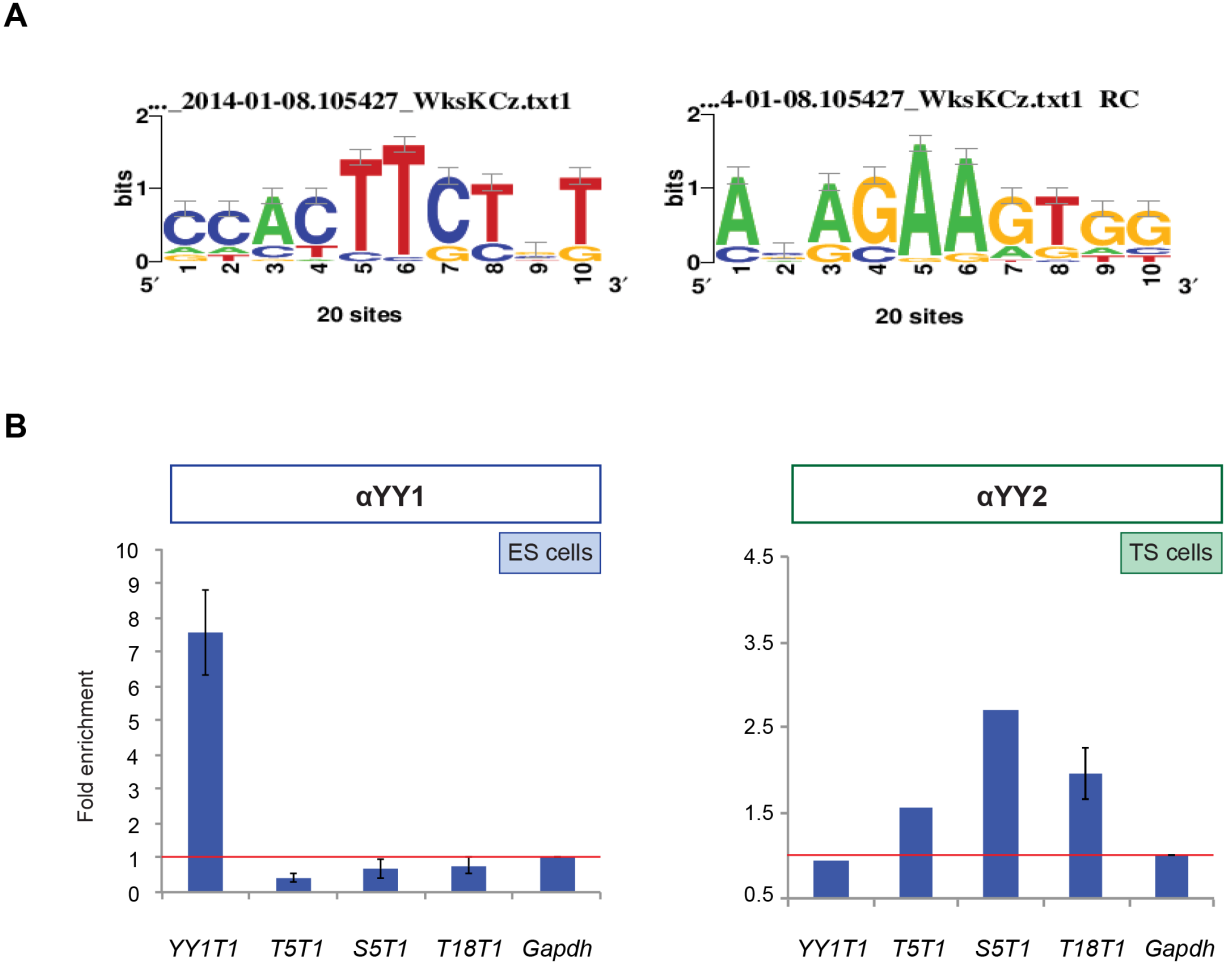
Association of YY1 and YY2 to chromatin. (A) A motif enriched in the genome wide YY2 ChIP-seq peaks is depicted as a sequence logo. Data from the top 20 peaks were analyzed as described in M&M. (B) Association of YY1 (in ES cells) or YY2 (in TS cells) to genomic targets was assessed as described in the legend to Fig 3B, using either αYY1 (left panel) or αYY2 serum (right panel). Codes for genomic targets analyzed refer to a YY1 genomic target (*YY1T1*) and several YY2 target genes (*T5T1*, *T18T1*) from Table 2 (see text for *S5T1*). The data in the left panel represent the average of three independent experiments ± SEM. The right panel shows the results of a typical experiment out of several performed, except for *T18T1*, multiple replicates performed.

All validated YY2 binding sites (Fig 3B) were taken from the top 20 binding sites according to enrichment score (Table 2). We searched the combined sequences in this list of 20 for a potential YY2 binding motif. Analysis revealed the presence of a consensus binding site ANAGAAGTGG (N indicating any residue), shown as a sequence logo in Fig 4A. This motif is present in all 20 sequences analyzed with a P-value ≤ 7.38e-04, (data not shown). Furthermore, this motif shares similarity with an AANATGG motif identified previously *in vitro* [1]. This data suggests that the sequences identified are bound directly by YY2 protein at a defined motif.

As YY2 shares high homology in the DNA binding zinc fingers with YY1 [1], we tested their mutual binding to several of the putative binding sites for YY2 identified in TS cells (Fig 4 and Table 2). In addition, a binding site *S5T1* with a rather low enrichment score (position 455) was also included as it obtained the maximum score when only reads mapping to multiple locations in the genome were taken into account. We carried out ChIP assays in combination with *locus*-specific qPCR as before, using either αYY2 or αYY1. Enrichment of particular *loci* was normalized against the *Gapdh* promoter (Fig 4B). As a control, we used a YY1 binding site identified in genome-wide location analysis of YY1 in ES cells [34]. Using αYY2 to corroborate YY2 binding, we calculated reproducible enrichment of several YY2 binding sites, including *T5T1*, *S5T1* and *T18T1*. In ES cell-derived chromatin immunoprecipitated using αYY1 (Fig 4B), we obtained a 7–8 fold enrichment of the *YY1T1* control *locus*, as opposed to the negligible enrichment of YY2 target sites. These results demonstrate mutually exclusive binding of YY1 and YY2, and suggest that *in vivo* binding sites for YY1 and YY2 differ, in line with subtle differences encountered in the consensus binding site *in vitro* [1, 3].

### Expression of putative YY2 targets as a function of *Yy2* levels

**Fig 5:**
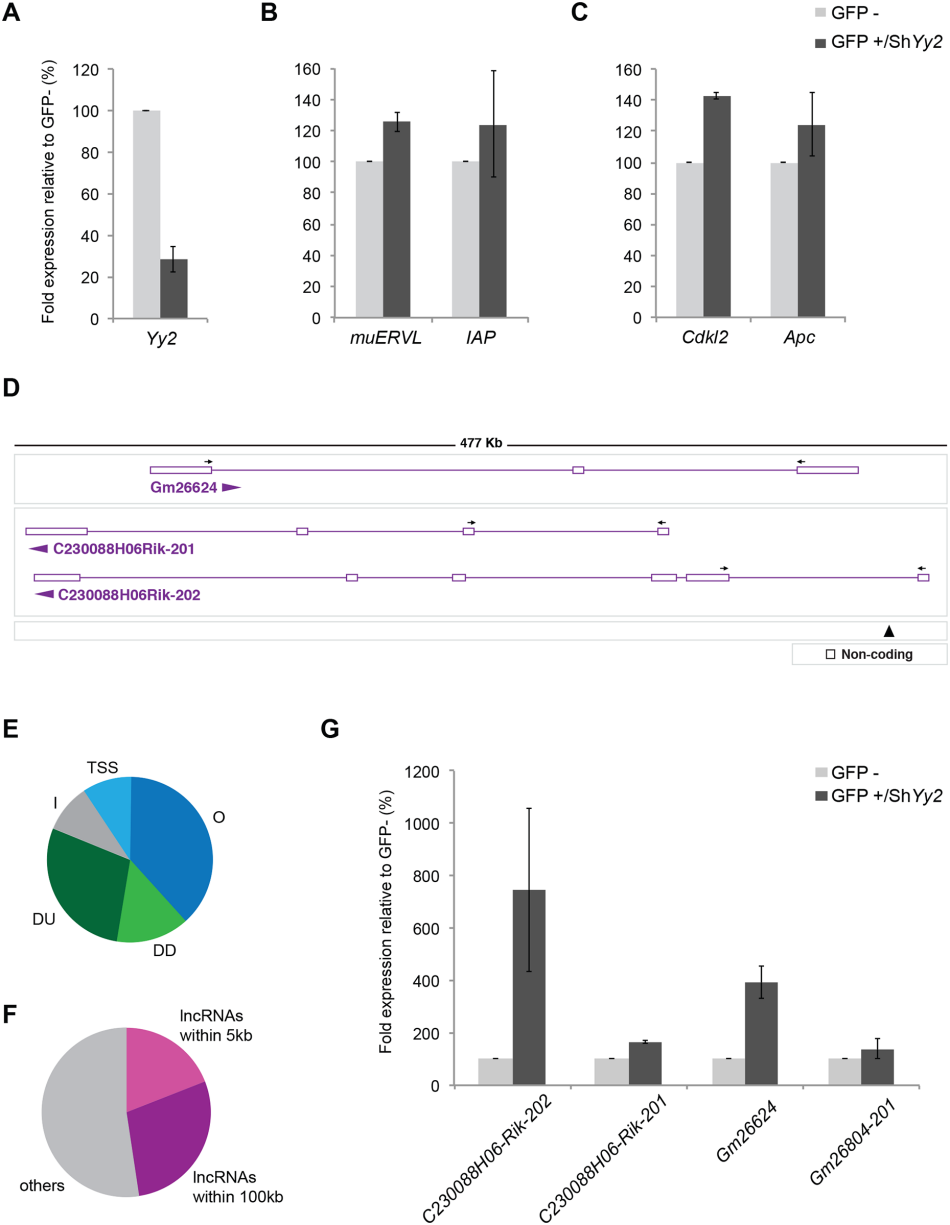
Expression analysis of genes associated with YY2 binding sites. (A-C) Gene expression levels in TS cells with attenuated *Yy2* levels. Cells were sorted by FACS after transfection with plasmids co-expressing shRNAs and GFP. Expression of either *Yy2* itself A, or of putative YY2 target genes B, C was compared between cells expressing a sh*Yy2* sequence (GFP+/Sh*Yy2*) and control cells (GFP-). All qPCR data were calculated using *Gapdh* as a reference gene and values are expressed relative to GFP-negative ES cells from the same experiment (100%). Data shown represent the average of two or more independent experiments ± SEM. No gene expression differences were observed between GFP positive and negative cells transfected with a construct carrying a non-specific shControl sequence. (D) Representation of a lncRNA locus analyzed on Chromosome 4 (GRCm38 Chr4:147018235–147491046). The primers utilized and the direction of transcription of the different transcripts is indicated with arrows and horizontal arrowheads, respectively. The location of the relevant YY2 peak number 2 (Table 2) is indicated with an arrowhead. (E) Localization of the 21 YY2 binding sites identified in TS cells. Peaks are grouped according to the location relative to transcribed annotated features in the database (NCBIM37).
TSS: within 2500 bp of a transcription start site;O: overlapping feature;DU: distal and upstream to TSS;DD: distal from TSS and downstream of transcribed feature;I: intergenic DNA at 100 kb from nearest annotated feature (F) Association of peaks with lncRNA genes. The graph shows the YY2 binding sites that map within 5 kilobases with respect to the nearest lncRNA gene (lncRNA within 5 kb), within 100 kilobases with respect to the nearest lncRNA gene (lncRNA within 100 kb) and other sites. (G) Expression levels of lncRNAs associated with YY2 binding sites (indicated in D) in *Yy2*-attenuated TS cells as described in A-C.

While YY2 selectively binds to IAP and MERVL retroviral elements in TS cells ([10]; Fig 2B), potential changes in the expression levels of these retrotransposons as a function of *Yy2* levels have not been addressed so far. We compared the expression levels of these elements between control TS cells and TS cells that carry shRNA-expressing vectors to attenuate *Yy2* expression levels. While *Yy2* levels were severely reduced under these conditions and represented only 20% of the levels detected in control cells (Fig 5A), we detected only minor differences between the expression levels of either *IAP* or *MERVL* in *Yy2*-depleted TS cells (Fig 5B).

Among the genes associated with YY2 peaks (Table 2) are the cell-cycle regulators *Cdkl2* (peak 4) and *Apc* (peak 19), with binding sites at 2.6 kb and 54.5 kb upstream of the Transcription Start Site (TSS), respectively. To establish a direct functional link between YY2 protein binding and gene regulation, we compared the mRNA expression levels of these genes in TS cells with attenuated levels of *Yy2*. We detected only small differences in expression levels of either protein-coding genes *Cdkl2* and *Apc* upon *Yy2* depletion (Fig 5C).

### *In vivo* YY2 binding sites linked to lncRNA genes

We surveyed the most significant YY2 binding sites (S2 Table and Fig 5E) for genes nearby. To the 20 peaks identified by the highest combined number of unique and repeated reads (Table 2) we added a site in the *Speer7-ps1 locus* (indicated as *S5T1*) that obtained the maximum score when only reads mapping to multiple locations in the genome were taken into account. Surprisingly, these binding sites were not confined to or preferentially located in gene promoters of protein-coding genes as might be expected [3, 7]. Only two of these 21 peaks mapped within kilobases (kb) of the nearest TSS (S2 Table and Fig 5E). Among the most significant peaks surveyed (S2 Table), several (eight) are located overlapping transcribed sequences, invariably far away from TSS (average distance around 70 kb). Other sites were located (far) downstream of transcribed genes (3/21), far upstream (6–60 kb, 6 sites) or more than 100 kb away from annotated features (2/21) (Fig 5E). Interestingly, four out of 21 binding sites are located either overlapping lncRNA genes or less than 5 kb away (S2 Table and Fig 5F). In addition, six more lncRNA genes are located within a 100 kb range of putative binding sites (Fig 5F). LncRNAs are a vast group of RNA molecules generally longer than 200 nucleotides that do not encode proteins [35–37]. They are polyadenylated, and may undergo post-transcriptional processing (5’ capping, splicing). While they are generally not highly expressed, they contribute to tissue- and cell-type specific as well as imprinted gene expression [36, 38].

In view of the prominent association of YY2 binding sites with lncRNA genes, we decided to include several of such genes in our analysis. Peak 2 (Table 2) overlaps three different lncRNAs transcribed in different directions (Fig 5D); peak 8 overlaps the transcribed region of a lncRNA named *Gm26804-201* (referred to as *Gm17279* in Table 2). We tested the expression levels of all these genes in *Yy2*-depleted TS cells. While the levels of *C230088H06-Rik-201* and *Gm26804-201* were independent of *Yy2* levels, the expression levels of *C230088H06-Rik-202* and *Gm26624* were significantly induced in the absence of *Yy2* (Fig 5G). These studies establish a functional link between YY2 protein binding and regulation in *cis* of expression of selected lncRNA genes.

## Conclusions

1. YY2 is expressed at high levels in two cell types representative of blastocyst stage embryos i.e. embryonic stem cells and in trophoblast stem cells.
2. YY2 binding to endogenous retroviral-like elements is not confined to TS cells and largely independent of REX1.
3. We have mapped *in vivo* YY2 binding sites in mouse (trophoblast stem) TS cells.
4. YY2 binding sites identified share a consensus binding motif. This motif is related to but different from reported *in vivo* binding sites for YY1.
5. Based on the analysis of a limited number of binding sites, YY2 association *in vivo* is distinct from YY1.
6. The expression levels of several lncRNAs linked to YY2 binding sites are moderately affected by *Yy2*-deficiency, suggesting regulation by YY2.

## Discussion

Consistent with the presence of YY2 in both ES and TS cell lines, we show that YY2 is associated *in vivo* with selected transposable retroviral elements (RE) in both cell types. Independent of the cell type assayed, we detected the highest levels of enrichment in ChIP assays for IAP (Fig 2). Although binding of the zinc finger domain of YY2 (as a GST-fusion) to a target *in vitro* was methylation-sensitive [1], IAP elements are reportedly methylated up to 90% in both ES [39] and TS cells [40]. The YY2 binding to IAP elements we observe might therefore be restricted to binding sites without GC dimers or to non-methylated copies of IAP. Despite the robust binding of YY2 to IAP elements we detected in both ES and TS cells (Fig 2), no changes in IAP levels were detected in cells with attenuated levels of *Yy2* (Fig 5B). Therefore, YY2 binding to IAP elements may serve to either prime posterior regulation, or be functionally secondary to other regulation mechanisms.

Apart from IAP, we also found a weaker association of YY2 to MalR/Orr1 elements (Fig 2), suggesting a potential role in their regulation. In addition to the role of YY1 in transcription of HERVK [41], YY1 was recently shown to be involved in the regulation of MMLV silencing in embryonic cells [42]. The new data we present here are consistent with the importance of YY1-family proteins in the epigenetic control of retroviral elements scattered over the genome in mammals, although no formal proof for YY2-dependent regulation of RE/ERV is available. It is still unclear whether the binding of YY2 to RE and IAP elements is cooperative, complementary or competing with REX1 and YY1. The absence of a major de-regulation in MERVL and IAP expression in *Yy2*-depleted TS cells could be due to overlapping functions of YY1-family members in regulation of RE, although binding affinities may be different for different factors and in different cell types.

We report novel YY2 binding sites in chromatin *in vivo*, identified as those sites most enriched in ChIP assays. We specifically assayed YY2 as opposed to YY1, as the YY2 serum used does not cross-react with YY1 (S2 and S3 Figs). Enrichment was determined after deep sequencing and compares reads identified in assays carried out using αYY2 serum with control assays using preimmune serum. The relatively low number of highly enriched sites (and the low probability scores associated) precludes the rigorous identification of genome-wide YY2 binding sites. Nevertheless, our approach method proved valid, as sites were easily confirmed in binding assays (Fig 3), and a consensus binding motif could be extracted (Fig 4A). Moreover, attenuation of *Yy2* levels in TS cells resulted in deregulated expression of at least a subset of genes associated with selected putative YY2 binding sites (Fig 5G). In HeLa cells, gene sets identified in expression microarrays that responded to *YY1* and *YY2* knock-down turned out to be largely overlapping [8]. Subtle differences were described however, as *YY1* controls more genes involved in proliferation, cytokinesis and DNA damage repair [7, 8], while attenuation of *YY2* levels reversed the YY1-depletion induced loss of proliferation and improved cell survival after UV damage [8]. No members of these gene sets appear in our analysis. We provide a list of novel putative YY2 binding sites, which may control genes not regulated by YY1. Whether these *loci* influence differentiation or tissue-specific gene expression in TS cells or in placental derivatives remains to be addressed by detailed functional analysis.

YY1 and its relative YY2 are mostly studied in the context of promoter regulation of target genes [3, 7], although a good portion (26,1%) of YY1 binding sites defined in mouse myoblasts reside in intergenic regions [43]. A survey of the 21 most significant YY2 binding sites identified in this manuscript (S2 Table; Fig 5E and 5F) suggested however that they were not confined to or preferentially located in gene promoters. It remains a possibility that YY2 is involved in long-range regulation as opposed to control of gene promoters. Alternatively, target genes in TS cells may differ from those in more differentiated cells with acquired tissue specificity. Initial searches for annotated features associated with putative YY2 binding sites in a range of 100 kb revealed the presence of protein-coding genes in 12 out of 21 cases (S2 Table). Within the same 100 kb range, ten out of 21 binding sites are associated with lncRNA genes (S2 Table and Fig 5F). Furthermore, expression analysis in TS cells with attenuated levels of *Yy2* revealed altered expression of selected lncRNA genes associated with binding peaks (Fig 5G). In light of our combined mapping and expression data, we suggest that YY2 might serve a more prominent role in regulation of non-protein coding genes. This hypothesis is not unprecedented, as YY1 frequently binds lncRNA genes in ES cells [38] and contributes to regulation of lncRNAs in skeletal myogenesis [35].

YY2 had been previously shown to bind *in vitro* to some but not all YY1 binding sites [3, 4]. Moreover, selection of duplex DNAs of randomized sequence for binding to zinc finger domains, yielded oligonucleotides with the same CGCCATNTT consensus motif for both YY1 and YY2 binding [1]. Based on this result and *in vitro* binding assays it was concluded that the overall DNA-binding patterns of YY1 and YY2 are similar. The putative YY2 binding sites reported here share a consensus motif ANAGAAGTGG, similar to but not identical to consensus motifs defined for YY1 *in vivo*. An AAGATGGCG motif was defined for cell-type-specific binding of YY1 [44]. We defined a consensus AAAATGGCTG for *in vivo* REX1 binding sites in mouse ES cells [33]. All such binding sites for YY1, YY2 and REX1 share the variant A/GAA/GG/ATGG(C) core. We would suggest therefore, that the small difference in the zinc finger region of each protein contributes to the preferred binding of either factor to a slightly distinct binding site. Of course, binding specificity may be fine-tuned by additional factors. The association with other DNA binding proteins co-determines binding affinity, allowing for unique interactions guided by the distinct amino termini of each protein [45]. The epigenetic status of the binding site may contribute, as already shown for methylation in case of YY1 [1]. Our data suggest that YY2 binding sites *in vivo* are distinct and separable from YY1 binding sites. The novel *in vivo* YY2 binding sites identified may form the basis for future studies aimed at defining unique functions of YY2.

## Supporting Information

S1 Fig Specificity of polyclonal serum towards YY2. (A) Western blots using αYY2 serum to detect GST-YY2 fusion protein, after pre-incubation with YY2 or REX1 fusion proteins, in the amounts indicated as molar excess. A band corresponding to GST-YY2 (GST-N-YY2) is marked with an arrowhead. Migration of molecular weight standards is indicated to the right. (B) Indirect immunofluorescence detection of YY2 (green) in confocal sections of 293T cells (Top panels) or HA-YY2 transfected 293T cells (lower panels). Nuclei were counterstained with DAPI as indicated, αYY2 staining was visualized using αRabbitBiotin followed by StreptavidinAlexa488. (C) Western blots to detect YY2 in cell lysates of E14T ES cells transfected with control shRNAs or shRNAs directed against *Yy2* (sh*Yy2*). The arrowhead indicates the YY2 protein, which migrates as a 60 kD protein. Migration of molecular weight standards is indicated to the left. Equivalent loading in each lane is demonstrated by stripping and reprobing the membrane with αTUBULIN antibodies.
(TIF)

S2 Fig The αYY2 serum does not immunoprecipitate or detect YY1. Alignment between YY2 and YY1 with the YY1 aminoacid sequence on top. Although YY2 shares good homology with YY1 in the Zinc Finger region (not shown), only limited similarity (32,4% identity) was found between the YY2 peptide used for antibody production (AA 1–173; the most C-terminal residue 173 is indicated with a black arrowhead) and the non-ZincFinger region of YY1.
(TIF)

S3 Fig Specificity of αYY2 serum. (A) and (B) The αYY2 serum specifically detects and immunoprecipitates YY2 as opposed to YY1. Human kidney 293T cells were co-transfected with plasmids expressing HA-tagged-intact YY1 or YY2 proteins. HA-YY1 or HA-YY2 were detected using αYY2 blot (B, top panel) or αHA blot (A and B, bottom panel), respectively, either directly in cell lysates (No IP) or after immunoprecipitation (IP) using αHA monoclonal antibody, αYY1 serum (Santa Cruz), αYY2 serum or PreI serum as indicated. Bands corresponding to HA-YY1/YY2 are marked with open (YY1) and closed (YY2) arrowheads, respectively. Bands corresponding to IgGs are indicated with arrows. Migration of molecular weight standards is indicated to the right (both A panels) and to the left in panels B. Dilutions used were as follows: αYY2 (1:14400) and αHA (1:100).
(TIF)

S4 Fig YY2 expression in ES and TS cells. YY2 expression in ES and TS cells assessed by indirect immunofluorescence. YY2 was detected with αYY2 and visualized using αRabbitBiotin followed by StrepAlexa488, nuclei were stained with DAPI (in blue). (A) Representative widefield images of YY2 in ES and TS cells acquired in a fluorescence microscope (40X objective). (B) Representative confocal sections (60X objective). In ES cell images 4, 5 and 6 and in TS cell image 6, 2X zoom was applied. ES and TS cell images were taken from 3 and 2 independent biological replicates, respectively.
(TIF)

S5 Fig Expression of *Mbtps2* and *Yy2* transcripts. (A) Representation of the *Mbtps2*-*Yy2 locus* and annotated transcripts, the arrow indicates the direction of transcription. The location of primer pairs used to amplify annotated *Yy2* transcripts (A-A, B-B and C-C) or annotated *Mbtps2* transcripts (D-D, E-E, F) is indicated. (B) Comparison of the amplification threshold of different primer pairs depicted in A, the E-F pair is Ef-Fr. Amplification was measured by quantitative RT-PCR as described in the M&M section. The amplification thresholds are represented as ΔCt values using *Gapdh* as a reference gene.
(TIF)

S1 Table Primers used. (XLS)

S2 Table Putative *in vivo* YY2 binding sites. Additional data on the most significant peaks identified (Table 2), and a site that obtained the maximum enrichment score when only reads mapping to multiple locations in the genome were taken into account (*S5T1*). The table lists each peak, and the distance to the TSS (in bp) of the nearest annotated feature (NCBI37/mm9) in the database within a range of 100 kb. Locations are arbitrarily classified as Distal (D, 2.5–100 kb away from TSS), Promoter (P, within 2.5 kb upstream of TSS), Overlapping transcribed sequences (O) or NF (Not Found). For those peaks overlapping with transcribed sequences, proximity to the relevant TSS is listed separately. The column “**TSS (~2.5 Kb)”** indicates which peaks qualify as promoter proximal. The presence of nearby lncRNAs is indicated in the last two columns. # This peak is localized 2352 bp downstream of the closest gene.
(PDF)
