# Supplementary figures and images for: In Vivo Chromatin Targets of the Transcription Factor Yin Yang 2 in Trophoblast Stem Cells
Source: PLoS One. 2016 May 18;11(5):e0154268. doi: 10.1371/journal.pone.0154268 (PMC4871433; doi:10.1371/journal.pone.0154268)

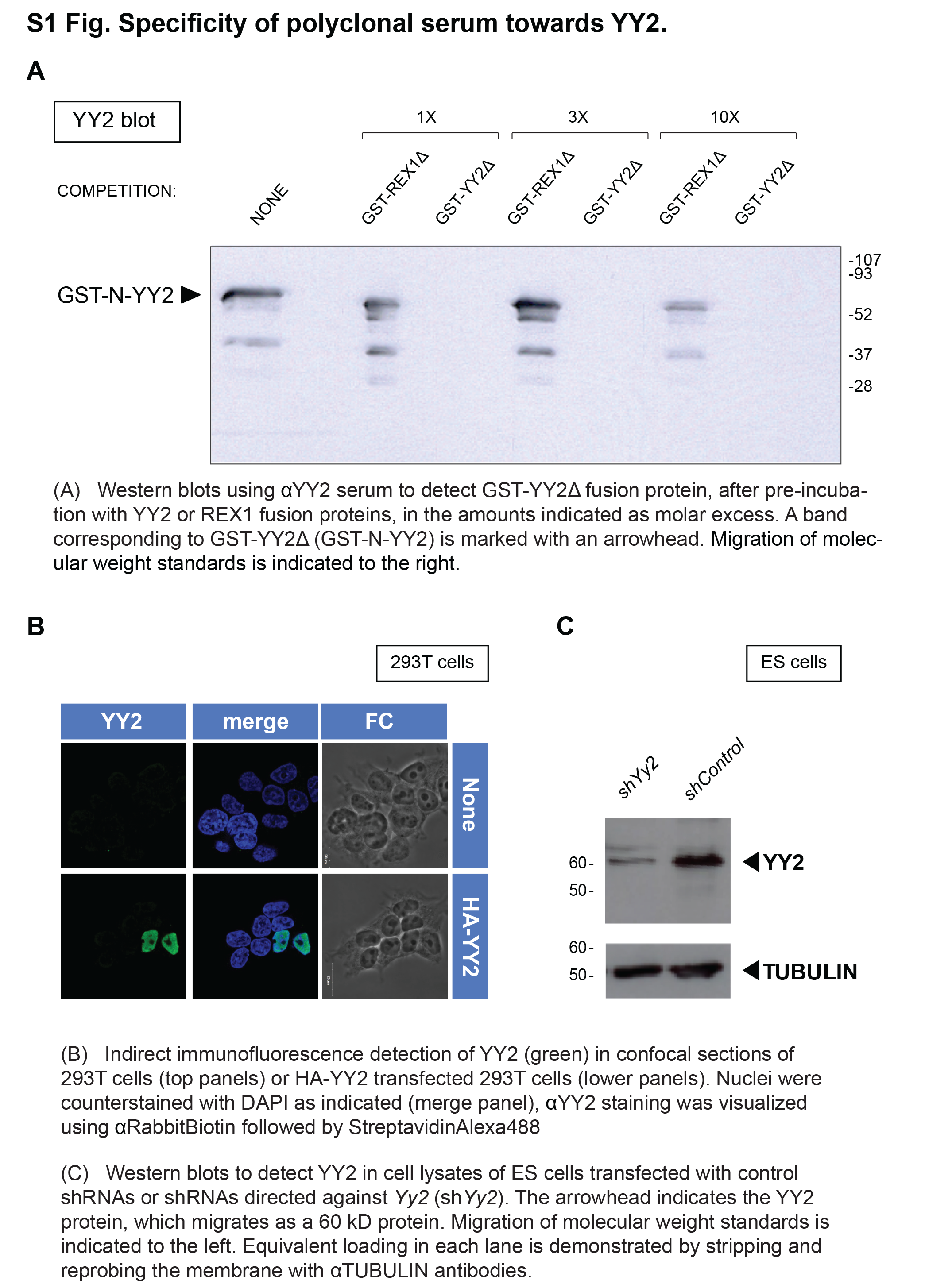

Supplement: S1 Fig — (A) Western blots using αYY2 serum to detect GST-YY2 fusion protein, after pre-incubation with YY2 or REX1 fusion proteins, in the amounts indicated as molar excess. A band corresponding to GST-YY2 (GST-N-YY2) is marked with an arrowhead. Migration of molecular weight standards is indicated to the right. (B) Indirect immunofluorescence detection of YY2 (green) in confocal sections of 293T cells (Top panels) or HA-YY2 transfected 293T cells (lower panels). Nuclei were counterstained with DAPI as indicated, αYY2 staining was visualized using αRabbitBiotin followed by StreptavidinAlexa488. (C) Western blots to detect YY2 in cell lysates of E14T ES cells transfected with control shRNAs or shRNAs directed against Yy2 (shYy2). The arrowhead indicates the YY2 protein, which migrates as a 60 kD protein. Migration of molecular weight standards is indicated to the left. Equivalent loading in each lane is demonstrated by stripping and reprobing the membrane with αTUBULIN antibodies. (TIF) [file pone.0154268.s001.tif]

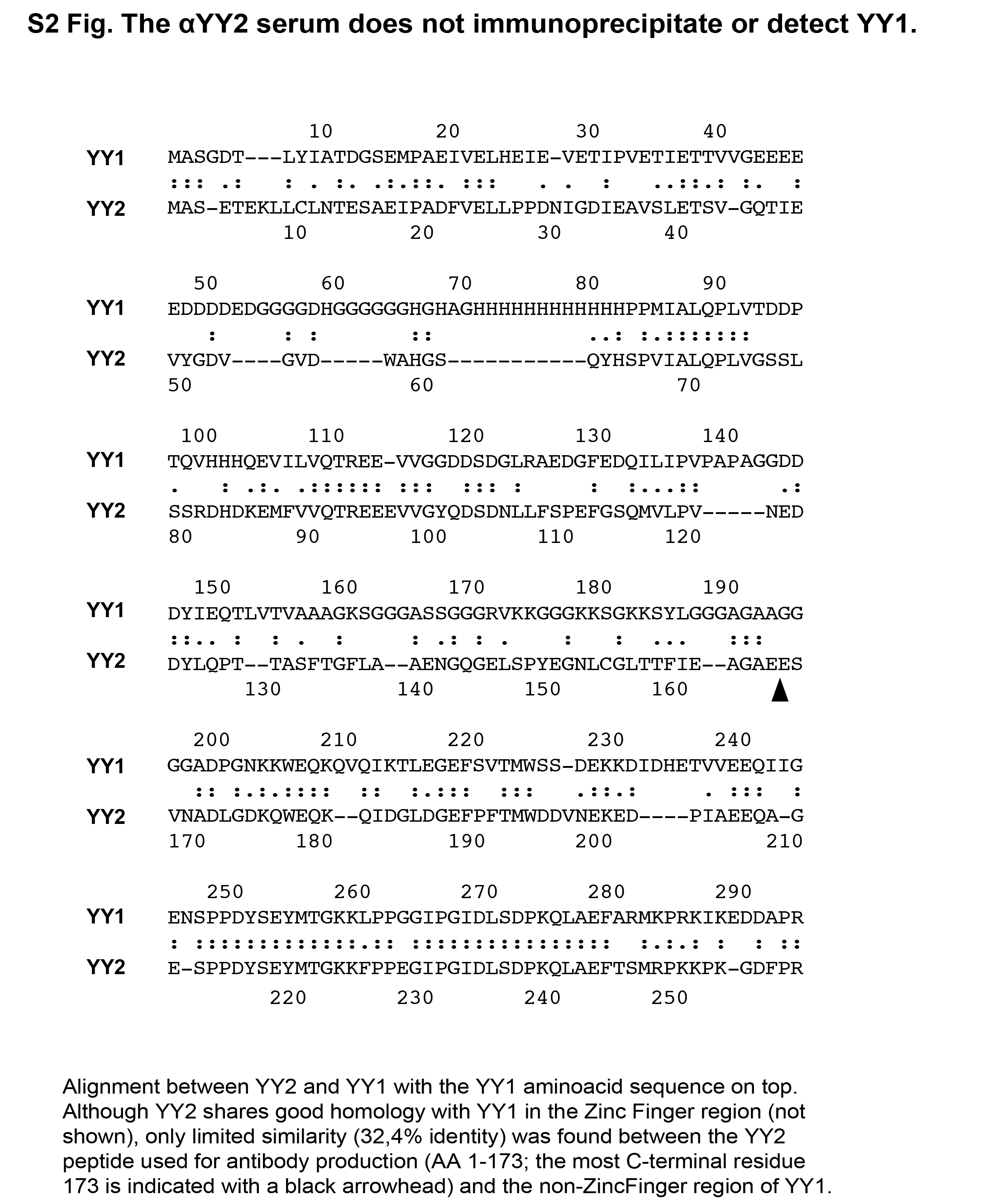

Supplement: S2 Fig — Alignment between YY2 and YY1 with the YY1 aminoacid sequence on top. Although YY2 shares good homology with YY1 in the Zinc Finger region (not shown), only limited similarity (32,4% identity) was found between the YY2 peptide used for antibody production (AA 1–173; the most C-terminal residue 173 is indicated with a black arrowhead) and the non-ZincFinger region of YY1. (TIF) [file pone.0154268.s002.tif]

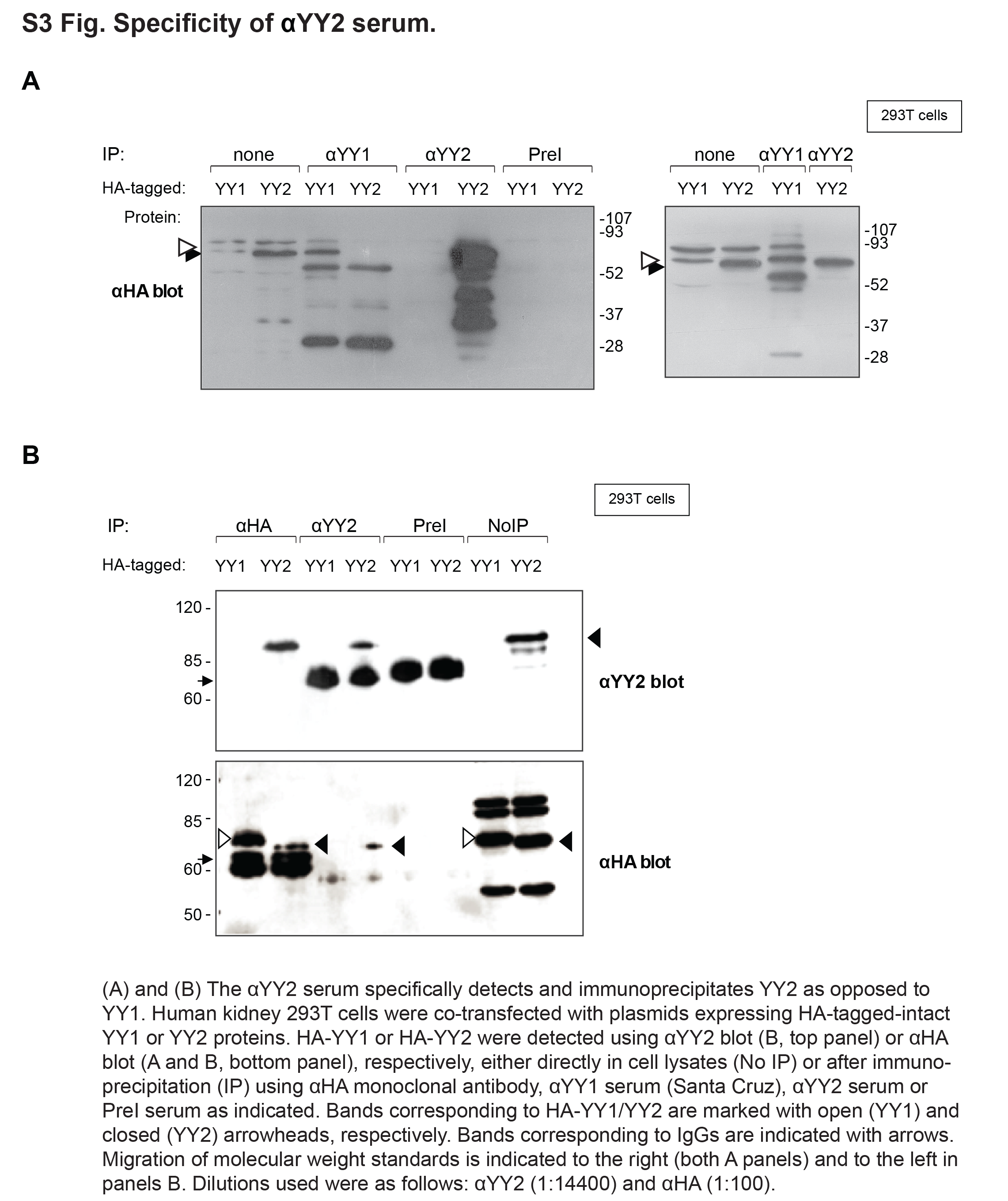

Supplement: S3 Fig — (A) and (B) The αYY2 serum specifically detects and immunoprecipitates YY2 as opposed to YY1. Human kidney 293T cells were co-transfected with plasmids expressing HA-tagged-intact YY1 or YY2 proteins. HA-YY1 or HA-YY2 were detected using αYY2 blot (B, top panel) or αHA blot (A and B, bottom panel), respectively, either directly in cell lysates (No IP) or after immunoprecipitation (IP) using αHA monoclonal antibody, αYY1 serum (Santa Cruz), αYY2 serum or PreI serum as indicated. Bands corresponding to HA-YY1/YY2 are marked with open (YY1) and closed (YY2) arrowheads, respectively. Bands corresponding to IgGs are indicated with arrows. Migration of molecular weight standards is indicated to the right (both A panels) and to the left in panels B. Dilutions used were as follows: αYY2 (1:14400) and αHA (1:100). (TIF) [file pone.0154268.s003.tif]

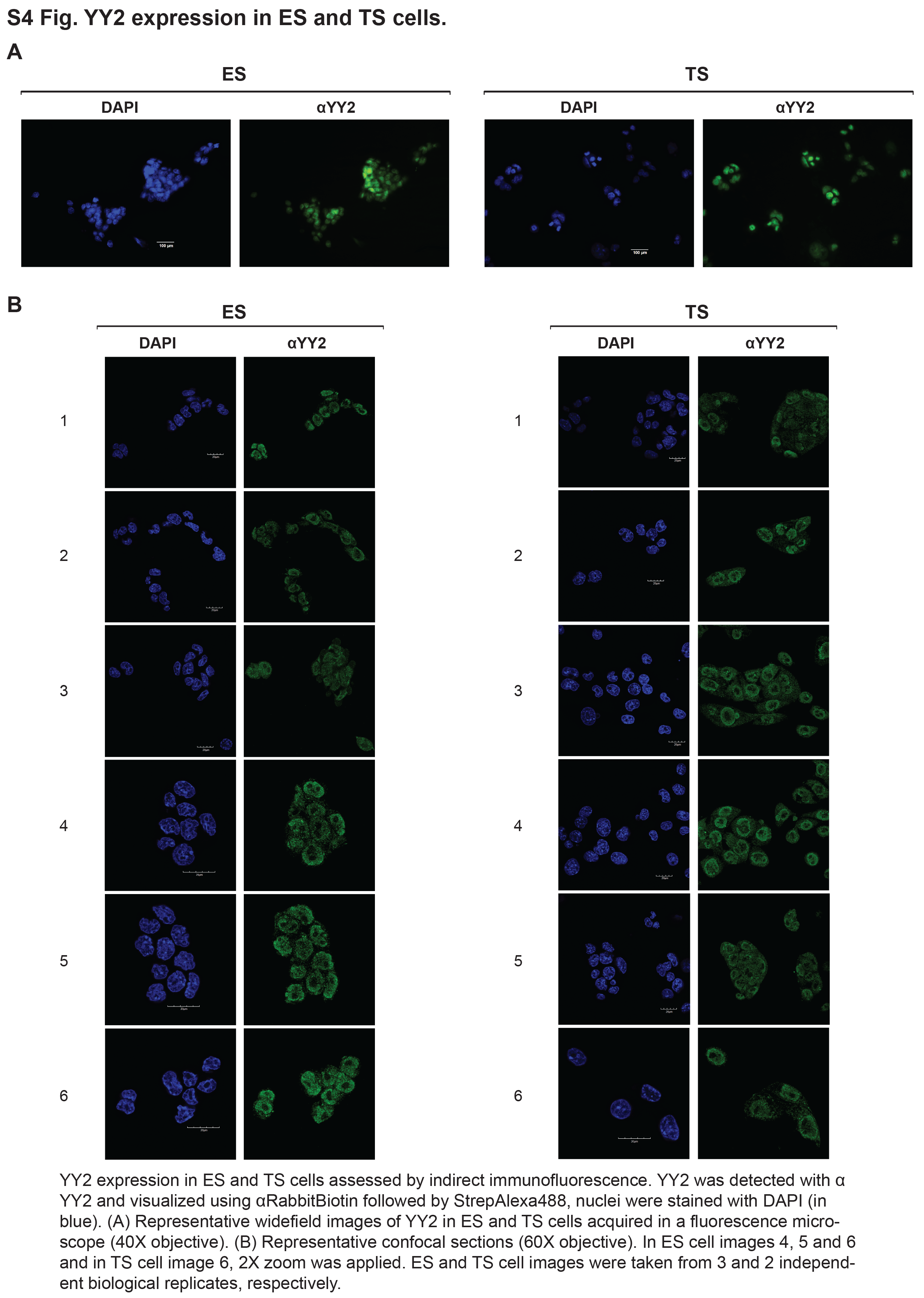

Supplement: S4 Fig — YY2 expression in ES and TS cells assessed by indirect immunofluorescence. YY2 was detected with αYY2 and visualized using αRabbitBiotin followed by StrepAlexa488, nuclei were stained with DAPI (in blue). (A) Representative widefield images of YY2 in ES and TS cells acquired in a fluorescence microscope (40X objective). (B) Representative confocal sections (60X objective). In ES cell images 4, 5 and 6 and in TS cell image 6, 2X zoom was applied. ES and TS cell images were taken from 3 and 2 independent biological replicates, respectively. (TIF) [file pone.0154268.s004.tif]

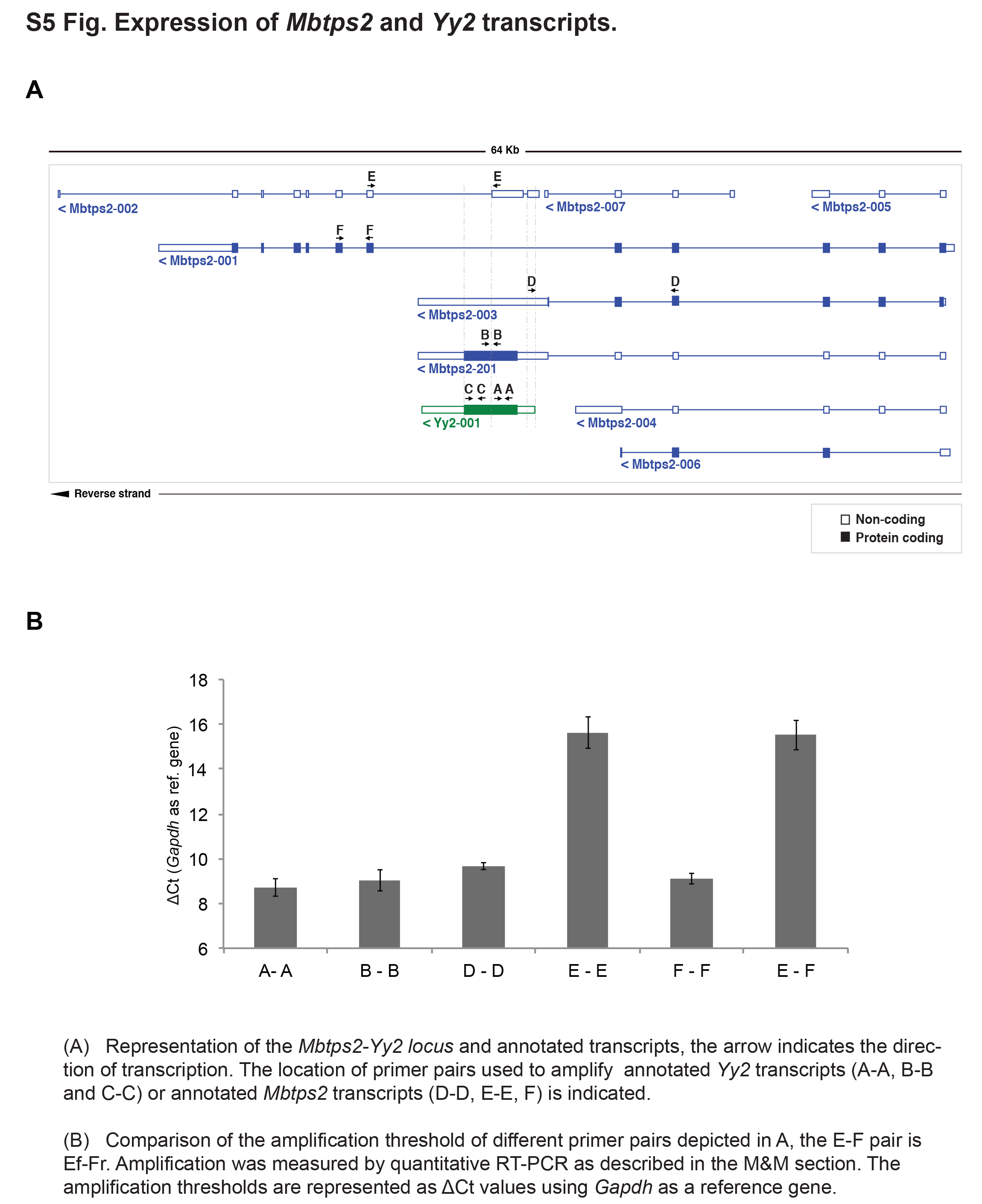

Supplement: S5 Fig — (A) Representation of the Mbtps2-Yy2 locus and annotated transcripts, the arrow indicates the direction of transcription. The location of primer pairs used to amplify annotated Yy2 transcripts (A-A, B-B and C-C) or annotated Mbtps2 transcripts (D-D, E-E, F) is indicated. (B) Comparison of the amplification threshold of different primer pairs depicted in A, the E-F pair is Ef-Fr. Amplification was measured by quantitative RT-PCR as described in the M&M section. The amplification thresholds are represented as ΔCt values using Gapdh as a reference gene. (TIF) [file pone.0154268.s005.tif]
