# Supplementary material for: In Vivo Chromatin Targets of the Transcription Factor Yin Yang 2 in Trophoblast Stem Cells
Source: PLoS One. 2016 May 18;11(5):e0154268. doi: 10.1371/journal.pone.0154268 (PMC4871433; doi:10.1371/journal.pone.0154268)
Supplement: S2 Table — Additional data on the most significant peaks identified (Table 2), and a site that obtained the maximum enrichment score when only reads mapping to multiple locations in the genome were taken into account (S5T1). The table lists each peak, and the distance to the TSS (in bp) of the nearest annotated feature (NCBI37/mm9) in the database within a range of 100 kb. Locations are arbitrarily classified as Distal (D, 2.5–100 kb away from TSS), Promoter (P, within 2.5 kb upstream of TSS), Overlapping transcribed sequences (O) or NF (Not Found). For those peaks overlapping with transcribed sequences, proximity to the relevant TSS is listed separately. The column “TSS (~2.5 Kb)” indicates which peaks qualify as promoter proximal. The presence of nearby lncRNAs is indicated in the last two columns. # This peak is localized 2352 bp downstream of the closest gene. (PDF) [file pone.0154268.s007.pdf]

Supplementary Table 2. Annotated features surrounding YY2 binding sites

**Peak Number**		**Distance to TSS of transcribed feature**			**TSS (~2.5 Kb)**	**LincRNA**	
						**Overlapping (5 Kb)**	**5 - 50 Kb distance**
**1**	D	51370					
**2**	O			70 kb	NO	Gm17317 and Gm16889	
**3**	D	6588					Gm17565
**4**	P		2600		YES		Gm9793
**5**	O			~ 15 Kb	NO		
**6**	NF						
**7**	O			> 100 Kb	NO		Gm15685
**8**	P		250 bp		YES	Gm17279 (250 bp)	
**9**	NF						
**10**	D	46749					
**11**	D	61453					Gm17278*
**12**	O			~ 65 Kb	NO		
**13**	O			~ 40 Kb	NO		
**14**	O			> 100 Kb	NO		
**15**	D	20364					Gm12027
**16**	O			~ 70 Kb	NO		Gm8378
**17**	O			~ 100 Kb	NO	A730081D07Rik	
**18**	D			> 100 Kb #			
**19**	D	22182					
**20**	D	7097					
**S5T1**	D	16721				Speer7-ps1	
